# The first plastid genome of a filamentous taxon ‘*Bangia*’ *sp*. OUCPT-01 in the Bangiales

**DOI:** 10.1038/s41598-018-29083-5

**Published:** 2018-07-16

**Authors:** Min Cao, Guiqi Bi, Yunxiang Mao, Guiyang Li, Fanna Kong

**Affiliations:** 1 Key Laboratory of Marine Genetics and Breeding (OUC), Ministry of Education, Qingdao, P. R. China; 2 Laboratory for Marine Biology and Biotechnology, Qingdao National Laboratory for Marine Science and Technology, Qingdao, China; 3 College of Marine Life Sciences, Ocean University of China, Qingdao, China; 4 Key Laboratory for Sustainable Utilization of Marine Fisheries Resources, Ministry of Agriculture, Yellow Sea Fisheries Research Institute, Chinese Academy of Fishery Sciences, 266003 Qingdao, China

## Abstract

Red algae are important primary photosynthetic organisms. The Bangiales comprise a morphologically diverse order of red algae. Until now, complete plastid genomes of the Bangiales were only mapped for foliose species. To date, no filamentous plastomes have been published. The aim of this study was to determine and analyze the complete plastid genome of the filamentous marine species *‘Bangia’* sp. OUCPT-01. It is a circular molecule, 196,913 bps in length with a guanine-cytosine (GC) content of 33.5%. It has a quadripartite structure with two single copy regions separated by two direct non-identical repeats. It has 205 protein-coding genes, 37 tRNAs, and 6 rRNAs. Therefore, it has a high coding capacity and is highly similar to other Bangiales species in terms of content and structure. In particular, it reveals that the genera in the Bangiales have highly conserved gene content and plastome synteny. This plastome and existing data provide insights into the phylogenetic relationships among the Bangiales genera of the Rhodophyta. According to its plastid- and mitochondrial genomes, *‘Bangia* 2*′* is a sister group to *Porphyra*. However, the position of *Wildemania schizophylla* in the Bangiales is still controversial. Our results show that the Bangiales divergence time was ~225 million years ago.

## Introduction

Red algae are an ancient lineage, which played a prominent role in the evolution of photosynthesis in eukaryotes^1^. The Bangiales is an important order of red algae whose members have simple and diverse morphologies. Earlier studies reported that the genus *Bangia* Lyngbye is a cosmopolitan taxon found in both marine- and freshwater habitats (http://www.algaebase.org). Nuclear small-subunit ribosomal RNA gene (nrSSU) revealed the phylogenetic relationships among the species in the Bangiales^2,3^. Sutherland *et al*.^4^ revised the order of the Bangiales based on global sampling and molecular analyses of *rbc*L and nrSSU. In this way, they provided new insights into the phylogenetic relationships among the taxa in the Bangiales. The freshwater species *Bangia atropurpurea* was confirmed to the genus *Bangia*. Numerous other taxa are currently referred to *B. fuscopurpurea*. It is still not known to which of these taxa the name is correctly applied^4^.

In 1990, a fossil of the well-preserved multicellular organism *Bangiomorpha pubescens* was discovered in rocks in the Hunting Formation of Somerset Island (Arctic Canada) dating from 1,250- to 750 million years ago (Ma). It strongly resembled modern *Bangia*^5^. Other fossil evidence of early organisms resembling *Bangia* was found in deposits dating from ca. 500 Ma^6^. These fossils provided evidence for eukaryotic sexual reproduction in *Bangia*-like organisms^7^. However, little is known about the timing of speciation in the Bangiales.

Plastids are vital organelles responsible for photosynthesis and the biosynthesis of starch, fatty acids, amino acids, pigments, and vitamins etc. They also participate in sulfur and nitrogen metabolic pathways^8^. Plastids have their own genomes and can replicate, transcribe, and translate^9,10^. Plastids may have arisen from a single primary endosymbiotic event in which a cyanobacterium penetrated a eukaryotic host cell^11,12^. The highly conservative nature and slow evolutionary rates of plastids make they suitable for molecular phylogeny and molecular ecology studies^1,13,14^. In recent years, the number of reports of completely mapped plastid genomes have substantially increased. To date, NCBI databases contain 41,000 new chloroplast sequences including forty-nine complete plastid genomes from red algal species^15–24^. Until now, complete Bangiales plastid genomes were only determined for foliose species. No plastid genomes of filamentous Bangiales species were published.

Here, we present the complete plastid genome of a filamentous Bangiales species along with its gene content and plastome architecture. We also explored the phylogenetic relationships and divergence time among the various Bangiales taxa by comparing their plastomes.

## Results

### Specimen identification

**Figure 1:**
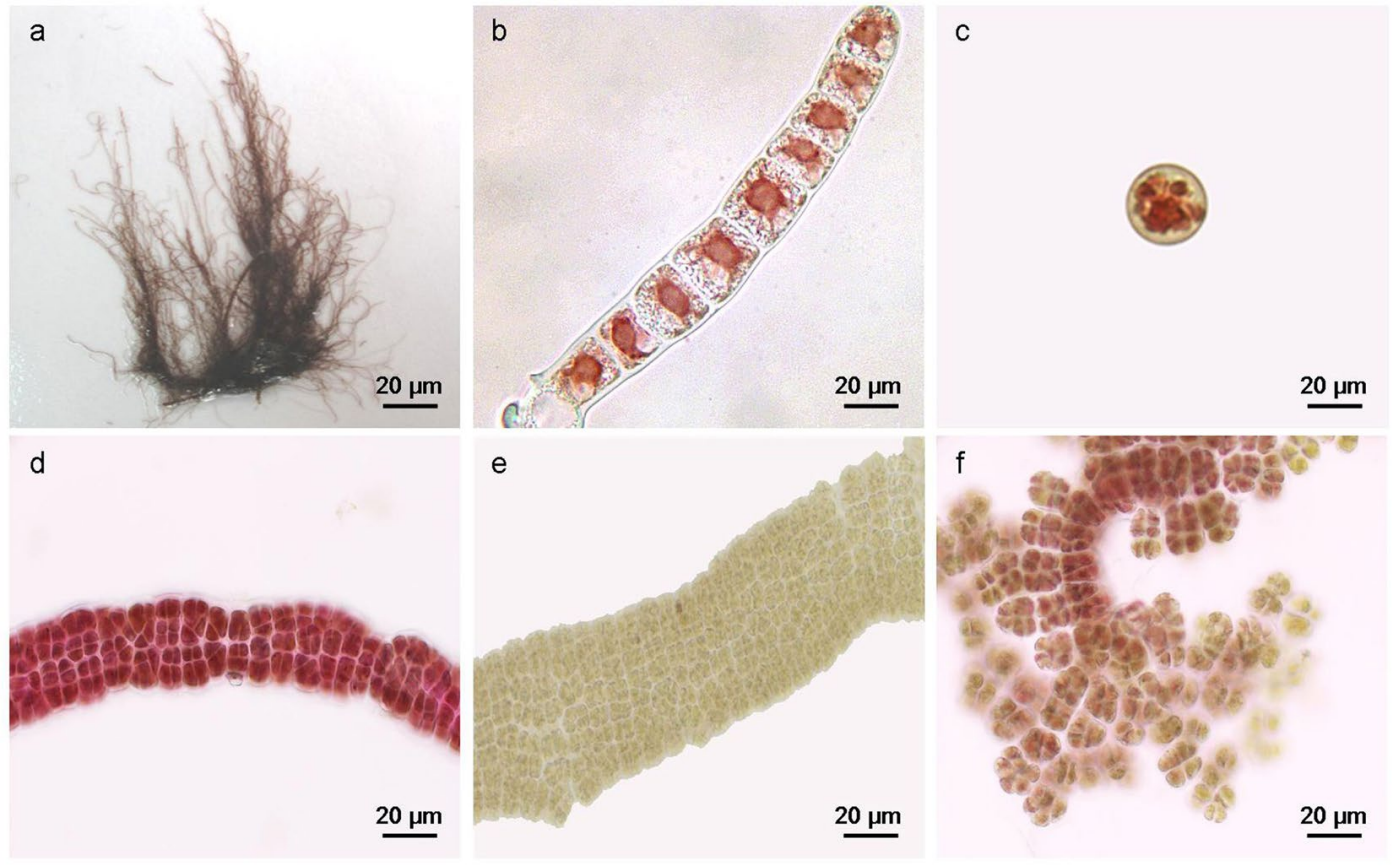
Microscopic structure of the filamentous bangialean *‘Bangia’ sp*. OUCPT-01. The thallus had uniseriate or multiseriate unbranched filaments composed of stacked, discoid cells enclosed in a sheath and containing stellate chloroplasts (**a**,**b**). The thallus releases archeospores (**c**) and forms reproductive cells by direct transformation of the vegetative cells (**d**–**f**).

The specimen studied here was identified as *‘Bangia’* sp. OUCPT-01 on the basis of its morphology and life history^25,26^. Briefly, the thallus consists of uniseriate or multiseriate unbranched filaments composed of stacked, discoid cells enclosed in a sheath. These cells have stellate chloroplasts (Fig. 1a,b). The reproductive structure form during the developmental process. The uniseriate unbranched filaments of the thallus may produce archeospores. After release, the archeospores undergo amoeboid movement and rapidly attach to the substratum. Their basal ends serve as multicellular rhizoid structures. The apical regions germinate into filamentous gametophytes. When the thallus grows into multiseriate filaments, some vegetative cells form spermatangia through serial division. Others may form carposporangia (Fig. 1c–e). The fertilized zygotosporangia divide mitotically to form zygotospores furtherly (Fig. 1f).

The concatenated nrSSU and *rbc*L genes from the *‘Bangia’* sp. OUCPT-01 together with the 160 species cited in Sutherland^4^ were used for phylogenetic tree reconstruction. The concatenated dataset consisted of 2,997 characters, of which 1,610 came from the nrSSU gene and 1,387 were derived from the *rbc*L gene. Phylogenetic topology demonstrated that *‘Bangia’* sp. OUCPT-01 formed a well-supported clade along with entities from New Zealand (BGA), Japan (CMNH UM BF1), and Taiwan (Taiwan). These findings were corroborated by maximum likelihood (ML) and Bayesian trees. The clade united “*Bangia 2*” and *Pyropia* then clustered with *Porphyra* (Figs S1 and S2).

### Sequencing and genome assembly

Two genomic DNA libraries (180 bp and 500 bp) were constructed for genome sequencing. Using Illumina sequencing technology, 28.37 Gb and 24.84 Gb of raw data were obtained, respectively. After adaptor trimming and quality filtering, a total of 48.89 Gb of clean data was used for contig assembly. Three linear contigs (134,139 bp, 17,085 bp, and 35,968 bp) of the plastid genome were screened out based on similarity matching to the reference plastid genome. The 5- and -3 terminal extensions of these linear contigs were generated by baiting and iteration and a circular plastid genome with a length of 196,913 bps was finally produced. The assembled genome was then compared with a total length of 65.8 kb (33.4%) generated by Sanger sequencing from 31 PCR amplicons of the plastid genome. The result showed that the difference rate was 1.98 × 10^−4^.

### Organization and gene content of the plastomes

**Figure 2:**
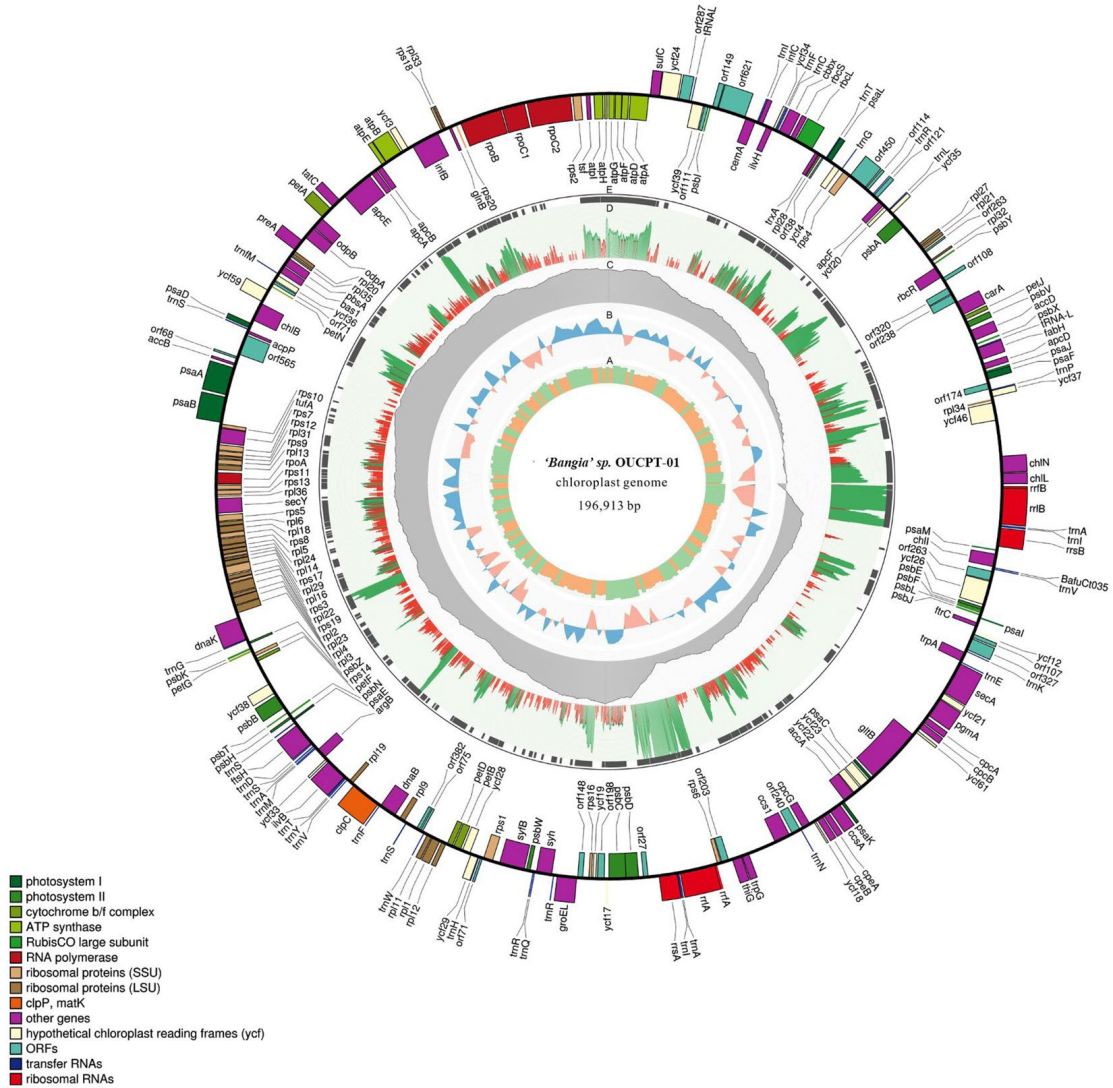
Gene map of the *‘Bangia’ sp*. OUCPT-01 plastid genome. Annotated genes are indicated in different colors. Genes inside the circles are transcribed clockwise whereas those outside the circles are transcribed counterclockwise.

The *‘Bangia’* sp. OUCPT-01 plastome was a typical circular DNA molecule 196,913 bps in length (Fig. 2). Overall, the GC content was 33.5%. The quadripartite structure consisted of a 151-kb LSC region and a 36-kb SSC region separated by two direct, non-identical repeats encoding 16S rRNA, 23S rRNA, 5 s rRNA, and two tRNA genes (*trnI* and *trnA*). The plastid genome included 205 protein-coding genes, 37 tRNAs, and 6 rRNAs (Table 1) representing 73.01%, 2.07%, and 4.88% of the total sequence, respectively. The gene-coding regions occupied 79.96% of the entire genome. There were 14 instances of overlapping genes (*psbC*-*psbD*, *atpD*-*atpF*, *ycf24*-*ycf*1*6*, *rps19*-*rpl*2, *rpl23*-*rpl4*, *carA-orf238*, and *rpl24-rpl14*). These properties indicated that it is a compact genome with high coding capacity.

**Table 1:** Characteristics of the *‘Bangia’* sp. OUCPT-01 plastome.

Total size [nt]	196,913
GC content	33.5%
Gene number	248
Protein genes	205
rRNA genes	6
tRNA genes	37
Protein-coding sequences [nt]	145,676 (73.01%)
tRNAs and rRNAs [nt]	10,830 (6.95%)

The *‘Bangia’* sp. OUCPT-01 plastome has an ancient gene repertoire according to its gene content, transcriptional regulators, and numbers of tRNA genes. A total of 156 protein-coding genes (except for unknown open reading frames (ORFs) were present in the plastome. They were homologous with the genes of the unicellular cyanobacterium *Synechocystis sp*. PCC 6803. The plastome contained almost all of the genes related to photosynthesis, phycobilisome formation, ATP synthesis, CO_2_ fixation, and electron transport systems. The gene components involved in photosynthesis included those participating in photosystem I (*psaA, B, C, D, E, F, I, L, M*, and *J*), photosystem II (*psbA*, *B*, *C*, *D*, *E*, *F*, *J*, *H*, *I*, *K*, *L*, *N*, *T*, *V*, *W*, *X*, *Y*, and *Z*), chlorophyll biosynthesis (*chlB, chlI, chlL*, and *chlN*), and phycobilisomes (*cpeA*, *apcA*, *B*, *D*, *E*, *F*, *cpcA*, *B*, and *G*). Genes involved in the biosynthesis of amino acids (*trpA and G*, *argB*, and *gltB*) and fatty acids (*accA*, *B*, and *D*) were also found in the plastome. Moreover, the plastome of *‘Bangia’* sp. OUCPT-01 retained many translation machinery and initiation factor genes including *infB*, *infC*, *tufA*, and *tsf*. The gene components also encoded RNA polymerase (*rpoA, rpoB, rpoC*_*1*_, and *rpoC*_*2*_), DNA replication (*dnaK* and *dnaB*), and organelle division (*ftsH* and *groEL*). In addition, the plastome coded for 37 tRNAs and contained two ribosomal RNA gene clusters (Supporting Information Table S1).

### Comparison of the *‘Bangia’* sp. OUCPT-01 plastome with those of other Bangiales

**Figure 3:**
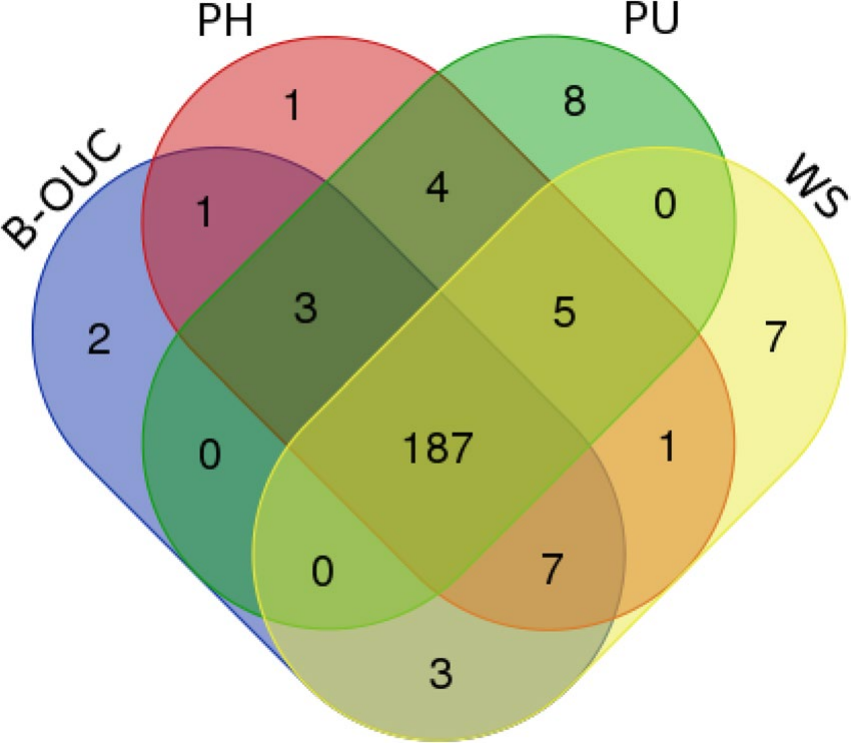
Venn diagram of genes gained and lost among the Bangiophyceae plastomes. PU, *Porphyraumbilicalis*; PH, *Pyropia haitanensis*; B-OUC, *‘Bangia’ sp*. OUCPT-01; WS, *Wildemania schizophylla*.

The gene contents of *‘Bangia’* sp. OUCPT-01 were compared with those of *Wildemania*, *Porphyra*, and *Pyropia*. A total of 187 genes were found to be common to them all (Fig. 3). They were mainly involved in the photosystem, translation, and transcription. Others were associated with ORFs of unknown function. In addition, some genes were found existing differencs among these species. For example, *sufC*, *bas1*, and *psbY* were present in *‘Bangia’* sp. OUCPT-01 and *W. schizophylla* but absent from *Py. haitanensis* and *P. umbilicalis*. Seven unidentified functional genes (*orf621, orf68, orf382, rpl29, orf287, orf111*, and *orf114*) were shared by *‘Bangia’* sp. OUCPT-01, *Py. Haitanensis*, and *W. schizophylla*. In addition, *orf62*, *ycf65*, *ycf31*, *orf58*, and *ftrB* were found in all of these species except for *‘Bangia’* sp. OUCPT-01. These comparisons demonstrated that the gene contents were highly conserved among the Bangiales species (Table S2) and that variations in the numbers of ORFs accounted for most of the differences among them.

**Figure 4:**
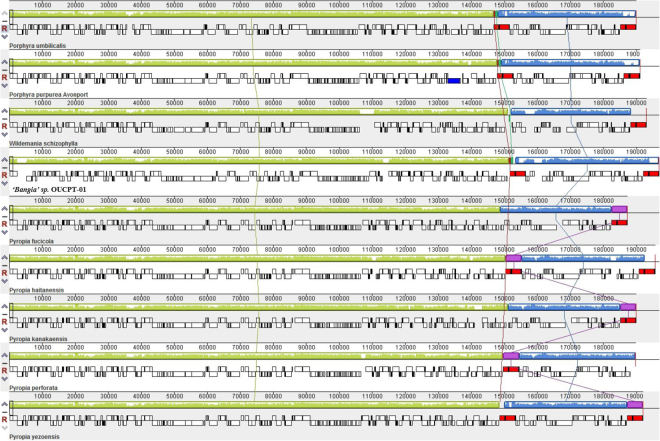
Genome comparison among Bangiophyceae plastid genomes. Two of the nine plastomes were highly co-linear. *Py. perforata* had one region representing ribosomal 23S, 16S, and 5S whereas the other species had two repeat regions (red modules).

The gene order of the nine Bangiales plastomes showed that, despite their evolutionary distance, the genomes are highly co-linear without any rearrangements (Fig. 4). Only one region representing ribosomal 23S, 16S, and 5S was found in *Py*. *perforata*. In contrast, repeat regions were presented in ‘*Bangia’* sp. OUCPT-01, *Porphyra*, and the other *Pyropia* species. This highly conserved synteny confirmed that plastid genome evolution was very gradual in the Bangiales.

### Weighted dN, dS, and dN/dS network analysis in the Bangiales

**Figure 5:**
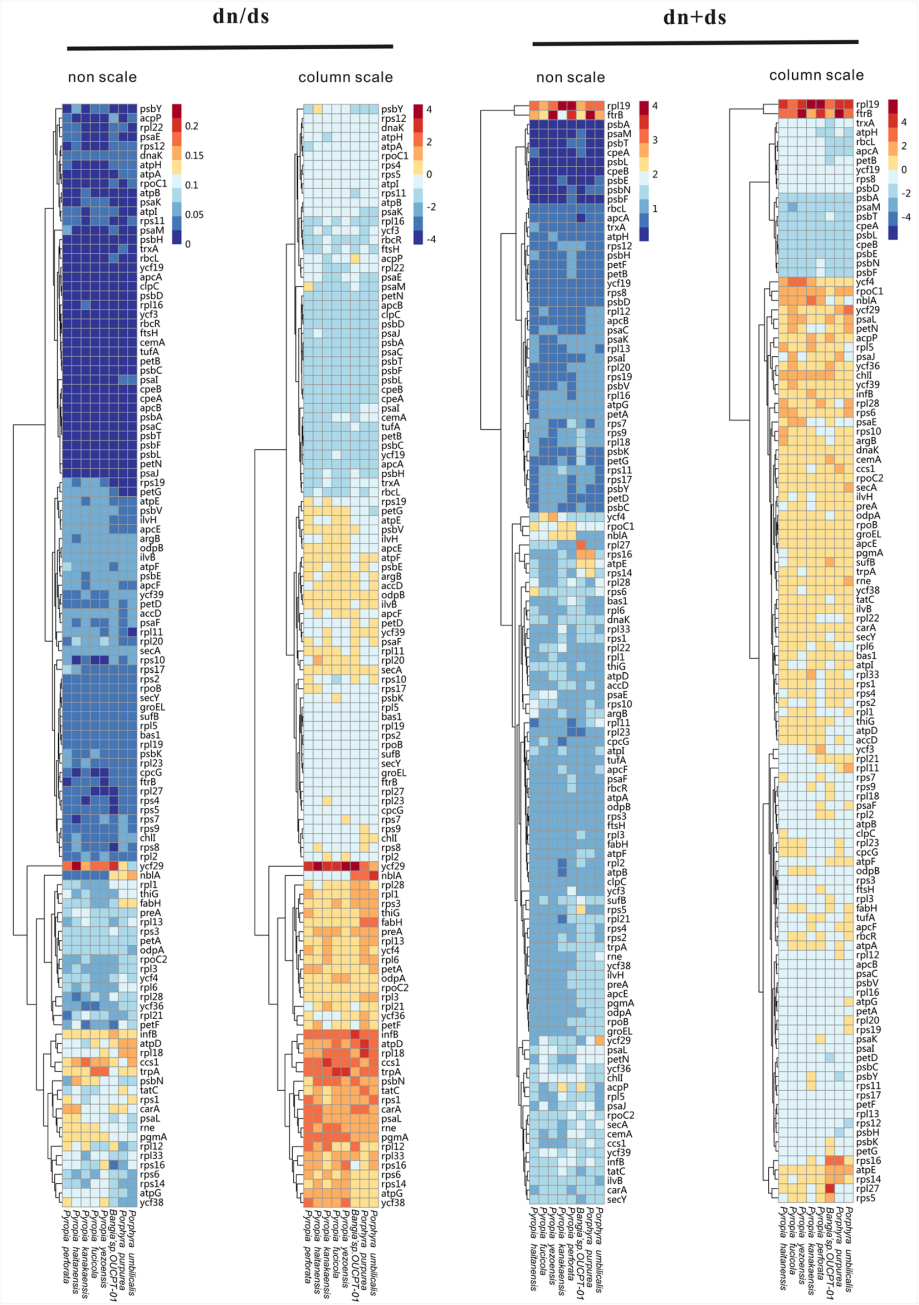
Weighted dN, dS, and dN/dS network analysis in the Bangiales. The figure on the left represents dN/dS and that on the right indicates dN + dS. Various modules are clustered together according to the relative similarities of their dN/dS and dN + dS expression patterns. For the dN/dS network, genes related to photosynthesis underwent strong selection pressure and are clustered in the deep blue module. Genes related to transcription initiation factors, ribosomal proteins and certain ycf-type transcription regulators are clustered in the deep orange module.

The ratio of dN to dS is an indicator of positive selection (>1) and purifying selection (<1). We compared dN/dS for each homologous group of genes from nine Bangiales species to determine whether there are selection pressure differences. *W. schizophylla* was selected as a reference. The mean ratios of dN to dS were very similar for all the coding sequences. It was difficult to separate them from each other. The dN/dS for *‘Bangia 2′* (0.051 ± 0.002) could not be distinguished from that of *Pyropia*. In *Pyropia*, it was as low as 0.049 (0.057–0.008). In *‘Bangia 2′*, it was as high as 0.053. The dN/dS of *‘Bangia 2′* was only very slightly lower than that of *Porphrya* (0.055 ± 0.001). The dN/dS ratios for all Bangiales species were <0.5 and varied little, indicating efficient purifying selection. The highest dN/dS ratios were observed for *ftrB*, *preA*, and *trpA* and were 0.20, 0.19, and 0.18, respectively. When we calculated the nonsynonymous and synonymous rates of *psaC*, *psbF*, *psbL*, and *psbT*, we found that the valuves of nonsynonymous of these genes were 0. That is to say, these genes are conservative among these species and did not occur nonsynonymous in their nucleotide sequences during their evolution process. The highest dN/dS ratios were detected in *rpl18* (0.12) and *rps1* (0.11) and the lowest in *rpl16* (0.01) and *rps12* (0.03) in the large ribosomal protein subunits. The dN/dS ratios of the photosynthesis genes were stable and ranged from 0 to 0.14. Weighted network analyses of dN, dS, and dN/dS showed that the genes related to photosynthesis were subjected to strong selection pressure and were clustered in the deep blue module (dN/dS). On the other hand, the genes related to the transcription initiation factor (*infB*), ribosomal proteins (*rpl18* and *rps14*), and certain ycf-type transcription regulators (*ycf29* and *ycf38*) were clustered in the deep orange module (dN + dS). Therefore, selection pressure on these genes were relatively low (Fig. 5).

### Phylogenetic relationship

**Figure 6:**
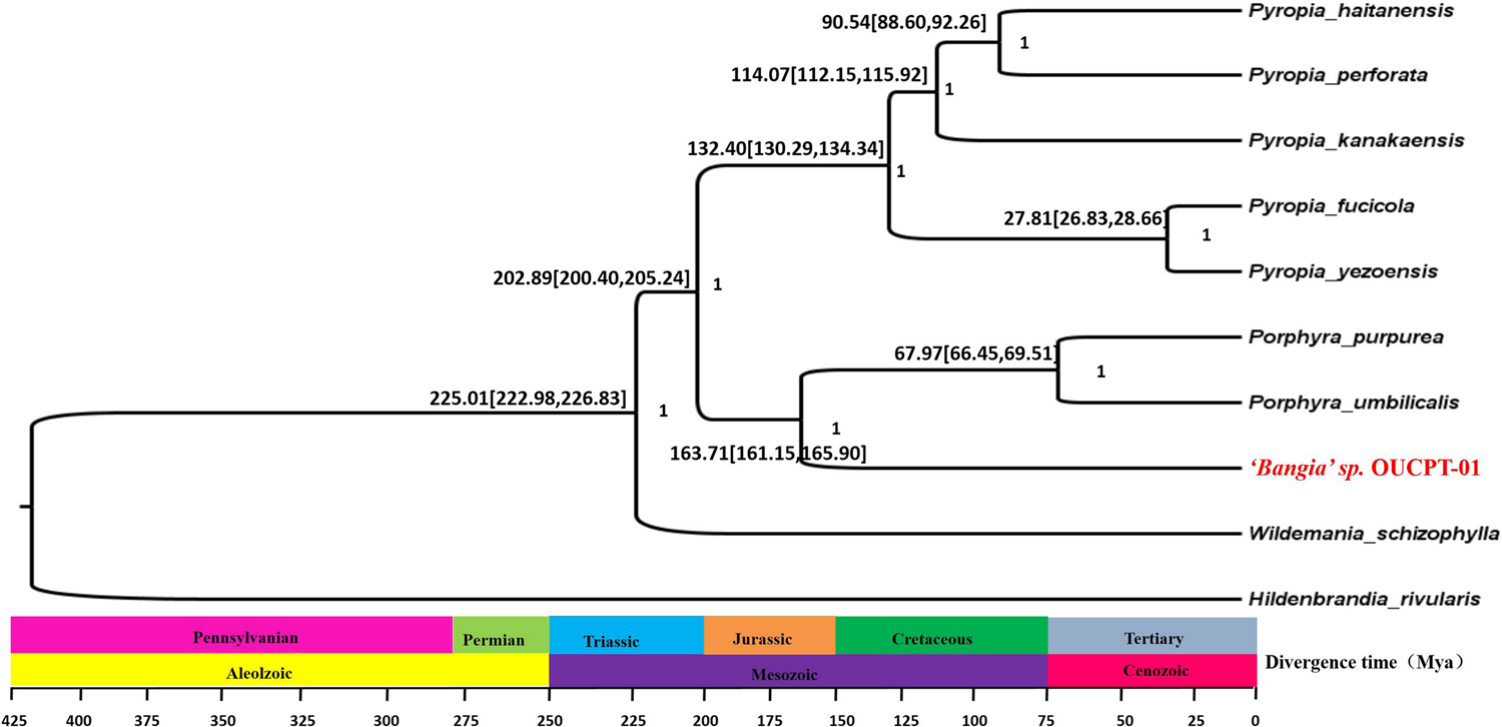
Chronogram showing Bayesian estimates of the divergence times among the Rhodophyta. Bottom: geologic time scale. The Bayesian posterior probabilities, the 95% confidence interval of the divergence time, and the divergence time are shown above the branches.

To elucidate the evolutionary position of *‘Bangia’* sp. OUCPT-01 in the Bangiales, we used different datasets to construct the phylogenetic tree. We extract the the amino acid and nucleic acid sequences from 153 common protein-coding genes from the plastid genomes of nine Bangiales species and one Florideophyceaen species (*Hildenbrandia rivularis* was selected as the outgroup). For the amino acid sequences, both Bayesian and ML trees were used to do the phylogenetic relationship analysis. The result showed that *‘Bangia’* sp. OUCPT-01 and *Porphyra* united together with 1.0 Bayesian posterior probabilities (BPP) (Fig. 6) then emerged as a sister group to *Pyropia* as it did in the ML tree with the 100% bootstrap value (Fig. S3). *W. schizophylla* appeared among the nine species with a long branch and distance (Fig. 6). Meanwhile, we constructed a phylogenetic tree with 18 mitochondrial protein-coding genes derived from other species in the Bangiales, the topology generated is congruent with the result from plastid genomes (Fig. S4). Besides, we constructed a phylogenetic tree using ML tree based on the nucleic acid sequence of 153 genes from plastid genomes. It showed that *W. schizophylla* is a sister to the clade of *Pyropia* taxa (Fig. S5).

### Divergence time analysis

We estimated the divergence times of various orders in the Rhodophyta using plastome consensus sequences. The constraints were chosen according to the divergence time between *P. yezoensis* and *Gracilaria tenuistipitata* (~900–1,000 Ma). Our relaxed molecular clock analysis suggested that the ancestor of the Florideophycidae originated in the mid-late Paleozoic no later than ~752 Ma (615–874 Ma). The major divergence events in this class included the emergence of the Gracilariales [305 (99–402) Ma], Gelidiales [155 (89–241) Ma], Halymeniales and Gigartinales [482 (358–604) Ma], Ceramiales [338 (252–448) Ma], Corallinales [515 (361–678) Ma], and Bangiales [224 (152–489) Ma] (Fig. S6). To test the time-estimate robustness for the Bangiales species, we used the 153 protein-coding genes from plastid genomes to estimate their divergence time. Our results showed that *W. schizophylla* diverged ~225 Ma (223–227 Ma) from the other Bangiales species. *‘Bangia’* sp. OUCPT-01 diverged from *Pyropia* ~203 Ma (200–205) and from *Porphyra* ~164 Ma (161–166 Ma). Plastid DNA of six species within *Pyropia* shared a common ancestor ~132 Ma (130–134 Ma). For the species in the genus *Porphyra, P. purpurea* and *P. umbilicalis* are separated from each other ~68 Ma (66–69 Ma) (Fig. 6).

## Discussion

### Plastome architecture and gene content

A comparison of plastomes indicated that the *‘Bangia’* sp. OUCPT-01 in this study was highly similar to *Pyropia* and *Porphyra* in terms of sizes, GC%, gene components, and structures. The highly congruent gene contents and conserved synteny confirmed that the plastomes in the Bangiales evolved slowly. Gene module comparisons showed high co-linearity among the plastomes of various Bangiales. Plastid genomes usually have a pair of inverted repeat (IR) regions^1^ containing genes encoding rRNAs and a variable number of tRNAs and proteins^27–31^. A quadripartite structure is a typical of plastome architecture in higher plants and green algae. The plastome is divided into four regions: two inverted repeats (IRa and IRb), one large single copy (LSC), and one small single copy (SSC). However, among the Bangiales, *Py. perforata* has one copy of the rRNAs coding region whereas there are two in *Py. fucicola*, *Py. haitanensis*, *Py. kanakaensis, Py. yezoensis, P. umbilicalis, P. purpurea, W. schizophylla*^15,20,23^, and *‘Bangia’* sp. OUCPT-01. The typical quadripartite structure has two repeat regions arranged in opposite directions. In contrast, two small direct non-identical repeats are found in Bangiales plastomes. The IR region may serve to increase the ribosomal component gene dosage^32^. The reason for this change in IR is unclear but some authors suggested that it is a convergent gene loss^15^. It is proposed, then, that the quadripartite structure is not conserved in the red algae and IRs may not be essential components of the algal plastid.

### Phylogenetic relationships of Bangiales taxa

Based on the concatenated nrSSU and *rbc*L dataset, *‘Bangia’* sp. OUCPT-01 was assigned to the *‘Bangia 2′* clade. The topology suggested that the phylogenetic relationship of *‘Bangia’* sp. OUCPT-01 to *Pyropia* is closer than its relationship to *Porphyra*. This observation corroborates the phylogenetic relationships reported by Sutherland^4^. However, a disparate topology is generated when complete plastomes were used to reconstruct the phylogenetic trees. The results from plastid genomes indicated that *‘Bangia’* sp. OUCPT-01 is more closely related to *Porphyra* than to *Pyropia*, which contradicts the results obtained from the phylogenetic analysis based on the concatenated nrSSU and *rbc*L gene dataset. With more and more organellar and nuclear genome data available, it has been a widespread phenomenon that researchers refer to use multiple genes to reconstruct phylogenies. However, the phenomenon of conflicting trees betwween multi-genes and single genes has accordingly become a difficult problem. It is increasingly understood that this difference may be caused by different datasets, number of taxon samplings and even the methods^33^. Different dataset substitutions may account for this discrepancy. In phylogenetic reconstruction, individual genes (mitochondrial, plastid, and nuclear) may yield independent estimates of the substitution parameters because of relative differences in their evolutionary rates. For example, the evolutionary rate of the *rbc*L gene is faster than that of 18S rDNA (ratio ~1.4)^34^. In general, datasets based on many genes or the entire genome can represent phylogenetic relationships more accurately than those derived from a single- or a few genes^35^. Concatenated protein sequences eliminate the differences caused by single gene substitution rates^36,37^. Thus, the selection of appropriate DNA fragments may influence the construction of phylogenetic relationships^38,39^. Also, the number of taxon sampling used may affect the accuracy of phylogenetic analyses because the contribution of each taxon may be different^40,41^. At present, seven filamentous- and eight foliose genera have been recognized in the Bangiales. Complete plastid genomes have been mapped for four of these. In our study, these four Bangiales genera were analyzed to construct a phylogenetic tree while Sutherland *et al*. used 161 species. The number of selected genes and taxon sampling are all different between our study’s and Sutherlands’, which may occupy the main reason for their different toplogy. Meanwhile, we found that our phylogenetic analysis based on the concatenated plastid genes (amino acids) and mitochondrial genes supported the theory that *W. schizophylla* appeared among the nine species with a long distance. This postulate was corroborated by Yang *et al*.^42^ while is also different from Sutherlands’. So, it is difficult to determine the exact position of *W. schizophylla* in the Bangiales on the currently available data. Therefore, to further ascertain and fully understand the phylogenetic relationship of *W. schizophylla*. and the other genus among the Bangiales, more genome data (including nucleus and organelles) and tanxon sampling from different taxa should be obtained in the futher work.

### Divergence time of Bangiales

Molecular clock methods incorporating plastid genome data may effectively date algal divergence^43^. We constructed phylogeny time trees for the Rhodophyta and the Bangiales based on the plastomes currently available. We estimated the divergence time of *Pyropia*, *Porphyra*, *‘Bangia’* sp. OUCPT-01 and *W. schizophylla* to be ~225 Ma, which is similar to that estimated using the *rbc*L gene^44^. The earliest record of the multicellular organism *Bangiomorpha pubescens* was dated to 1,250 Ma. This species strongly resembled the modern red algae *Bangia*. The filamentous *‘Bangia’* sp. OUCPT-01, then, is a relatively modern species. *‘Bangia’* sp. OUCPT-01 may have diverged from *Pyropia ~*202 Ma and from *Porphyra ~*163 Ma. These divergence times are consistent with that of *Bangia atropurpurea* and *Porphyra* (150 Ma)^45^. According to our molecular markers, the divergence time of the Bangiales coincided with the Mesozoic era and was congruent with the appearance of various algae at that time.

Our study provided a better understanding of the phylogenetic position and the divergence time of the Bangiales. Complete plastome mapping for other bangialean species will enhance the accuracy of the estimation the divergence time of Bangiales. It will also elucidate the origin, evolution, and adaptation of the Rhodophyta.

## Materials and Methods

### Seaweed culture

*‘Bangia’ sp*. OUCPT-01 gametophytes were collected from a farming raft in Putian, Fujian Province, China. The sampling site was located at N25°13′38.54″ and E119°28′9.87″. After removing the epiphytes and rinsing the samples several times with sterilized seawater, the gametophytes were cultured in a laboratory. They were grown at 20 ± 1 °C under fluorescent light (12 h:12 h photoperiod; 60 μmol photons·m^−2^·s^−1^ light intensity) and with constant aeration in sterilized filtered seawater supplemented with Provasoli-Enriched Medium^46^. The culture medium was replaced every 5 days. We also have made a voucher specimen for the *‘Bangia’ sp*. OUCPT-01 in our lab in order to do some identification work in the future using the same materials.

### Specimen identification

Specimen morphology was examined and photographed with an Olympus BX51 microscope (Olympus Corp., Tokyo, Japan). A concatenated nrSSU and *rbc*L gene dataset was used for molecular identification. The primers (5′AAATGGGTTACTGGGATG 3′ and 5′ GCTTTATTTACGCCTTCC 3′) were used to amplify the *rbc*L gene. The amplification conditions were: pre-denaturation at 95 °C for 5 min; 35 cycles with denaturation for 1 min at 95 °C; primer annealing at 50 °C for 1 min; extension for 2 min at 72 °C; and final extension for 10 min at 72 °C. The reaction volume was 20 µL and consisted of 2 µL genomic DNA, 0.6 µL dNTP (10 mM), 0.2 µL of each primer (10 µM), 5.2 µL distilled water, and 10 µL reaction buffer with 2 units *LA taq* (TaKaRa Bio Inc., Kusatsu, Shiga, Japan). The primers (5′CGATTCCGGAGAGGGAGCCTG 3′ and 5′ CTT GTTACGACTTCTCCTTCC 3′)^47^ were used to amplify the nrSSU gene. The reaction conditions were the same as those for *rbc*L except the annealing temperature was 56 °C. All PCR products were sequenced by the Sanger method. The nrSSU- and *rbc*L genes of *‘Bangia’* sp. OUCPT-01 were deposited in Genbank (nrSSU: KP747608; *rbc*L: KP747609). Sequences of nrSSU and *rbc*L genes from other filamentous bangialeans (*Pyropia* and *Porphyra*) were obtained from Sutherland *et al*. (Supporting Information Table S3). The common sequences were direction-adjusted then aligned using MEGA v. 6.0^48^. The optimal evolutionary model for the dataset was determined with jModelTest^49^. ML and Bayesian phylogenetic trees were plotted with RAxML v. 7.2.2^50^ and MrBayes v. 3.2^51^ using the GTR + I + G model. Branch confidence levels were estimated based on 1,000 bootstrap replications for the ML tree. The Bayesian inference was performed using a general time-reversible GTR + I + G model with an invariant site proportion of 0.46 and a gamma distribution shape parameter of 0.70. Four simultaneous chains were run for 10,000,000 generations. The initial 10% were discarded as burn-in. The dataset is available in Supporting Information Data 1.

### DNA sequencing and assembly

Samples were collected manually, rinsed with sterile seawater, then dried with filter paper. A total of 6 μg genomic DNA was isolated using CTAB buffer^52^. DNA purity was tested using a NanoPhotometer^®^ spectrophotometer (Implen, Inc., Westlake Village, CA, USA). The DNA concentration was measured with the Qubit^®^ DNA Assay Kit and the Qubit^®^ 2.0 Fluorometer (Life Technologies Corp., Carlsbad, CA, USA). Sequencing libraries were generated using an IlluminaTruseq^TM^ DNA Sample Preparation Kit (Illumina, San Diego, CA, USA) following the manufacturer’s recommendations and sequenced on the Hiseq2000 sequencing platform with a TruSeq SBS Kit v. 5 (Illumina, San Diego, CA, USA) in a 100 × 100 bp paired-end run. The raw data were adapter- and quality-trimmed (error threshold = 0.05; n ambiguities = 5; sequence length ≤ 50 bp). Clean reads from genome surveying were assembled using SOAPdenovo (Short Oligonucleotide Analysis Package) with its default settings^53^. The assembled contigs were blasted against the reference plastid genome (*Pyropia haitanensis*: NC_021189) using BLAST (http://blast.ncbi.nlm.nih.gov/; conditions: query coverage ≥70%; E-value ≤ 1e-10). After assembly and blasting, the plastid-related contigs of *‘Bangia’* sp. OUCPT-01 were screened out. To close the gaps between them, each contig was extended in both the 5′ and the 3′ directions using baiting and iteration^54^. To evaluate the accuracy of the plastid genome, 31 pairs of primers (Supporting Information Table S4) were randomly designed from it and confirmed by the Sanger method. The PCR sequences were aligned to the assembled plastid genomes with MEGA v. 6.0^48^ and used to validate the accuracy of the plastid genome sequence. The final *‘Bangia’* sp. OUCPT-01 version was deposited to Genbank (Accession number: KP714733).

### Plastid genome annotation and analysis

The protein-coding genes and the putative open reading frames (ORFs) of the plastid genome were annotated using DOGMA^55^ coupled with the NCBI ORF-finder (http://www.ncbi.nlm.nih.gov/gorf/gorf.html). BlastX and BlastN searches were performed at NCBI (http://www.ncbi.nlm.nih.gov/BLAST/). The tRNA genes were identified using tRNA scan-SE v. 1.21 (http://lowelab.ucsc.edu/tRNAscan-SE/) and the Mito/Chloroplast model. The ribosomal RNA genes were identified using the RNAmmer 1.2 Server (http://www.cbs.dtu.dk/services /RNAmmer/). A graphical representation of the annotated genome was produced with OGDRAW (http://ogdraw.mpimp-golm.mpg.de/cgi-bin/ogdraw.pl). Four Bangiales species (*Wildemania schizophylla*, *Pyropia haitanensis*, *Porphyra umbilicalis*, and *‘Bangia’* sp. OUCPT-01) were selected to compare the gene contents with a Venn Diagram (http://bioinformatics.psb.ugent.be/webtools/Venn/). Nine completed Bangiales plastid genomes (*P. purpurea*, *P. umbilicalis*, *Py. haitanensis*, *Py. yezoensis*, *Py. perforata*, *Py. kanakaensis*, *Py. fucicola, W. schizophylla*, and *‘Bangia’* sp. OUCPT-01) were selected for structural comparisons. Plastid genomes were aligned with the Mauve Genome Alignment program using its default settings^56^.

Evolutionary selection in the genes common to the Bangiales was estimated using nonsynonymous (dN) substitution, synonymous (dS) substitution, and their ratios (dN/dS). They were calculated for: (a) large- and small subunits of the ribosomal protein-coding genes, (b) genes related to photosynthesis, and (c) groups of genes with the other functions. Each group of homologous protein sequences was aligned using MEGA v. 6.0^48^. Columns with gaps were removed from the amino acid alignments. Pairwise nucleotide alignments were obtained by directly retrieving the sequences in the homology group alignment. The dN, dS, and dN/dS values were calculated using DnaSP v. 5^57^. The weighted network of dN, dS, and dN/dS in the Bangiales was drawn by R script.

### Phylogenetic analyses and divergence time estimates

To elucidate the evolutionary position of *‘Bangia’* sp. OUCPT-01 in the Bangiales, we extract common genes from nine Bangiales species and an outgroup taxon (*H. rivularis*, a Florideophyte). For the genes extraction, we put the genbank formats of nine Bangiales species and *H. rivularis* together, then we run the perl script (get_homologues.pl) to extract their common genes according to the blast results. Finally, we extracted 153 common protein-coding genes (the amino acid and nucleic acid sequences) from these plastid genomes (Table S5). Subsequently, the genes were individually aligned using MEGA v. 6.0^48^. After we aligned these genes one by one, we pasted all the genes according to their gene names alphabetical order. Finally, we got different matrix (Supporting Information Data 2 for the amino acids and Supporting Information Data 3 for the nucleic acids). The optimal evolutionary model for the dataset was determined by jModeltest^58^. The best models for the amino acids and the nucleic acids are the cpREV and GTR + I + G model, respectively. For the GTR + I + G model, the proportion of invariant sites was 0.2760 and the gamma distribution shape parameter was 0.4980. Four simultaneous chains were run for 10,000,000 generations. The initial 10% were discarded as burn-in. Ninety-five percent confidence intervals on the divergence dates were calculated in log-likelihood units around the estimates^59,60^.

To calculate the divergence time of the species in the Bangiales, we first calculated the divergence time of the species in the Rhodophyta. Firstly, the divergence time of *Py. yezoensis* and *Gracilaria tenuistipitata* (900–1,000 Ma) calculated from eight fossils was selected as time scale for the species of the Rhodophyta^61^. The nucleotide sequence of 67 genes common to twenty-six Rhodophyte species and an outgroup species (*Cyanophora paradoxa*) were used to estimate the divergence time of the various orders in the Rhodophyta (Supporting Information Data 4, Table S6). The extraction method was same as mentioned above. Bayesian inference was performed using MrBayes v. 3.2^51^ and a GTR + I + G model. The same method was used as that described above except the invariant site proportion was 0.3240 and the gamma distribution shape parameter was 0.6890. The mean divergence time of *Py. yezoensis* and *G. tenuistipitata* was set to 950 Ma and the variance of the normal distribution was fixed at 25. After the divergence time of the red algal orders was estimated, we used 153 genes common to plastid genomes from nine Bangiales species and an outgroup species (*H. rivularis*) to estimate the divergence time of species in the Bangiales. The same method was used as that described above. The mean divergence time of *Py. yezoensis* and *Py. fucicola* was set to 27 Ma and the variance of the normal distribution was fixed at 1.

## Electronic supplementary material

Supporting Information

Dataset
