# Supplementary material for: The first plastid genome of a filamentous taxon ‘Bangia’ sp. OUCPT-01 in the Bangiales
Source: Sci Rep. 2018 Jul 16;8:10688. doi: 10.1038/s41598-018-29083-5 (PMC6048033; doi:10.1038/s41598-018-29083-5)
Supplement: Supplementary file 1 — Supporting Information [file 41598_2018_29083_MOESM1_ESM.pdf]

The first plastid genome of a filamentous taxon *'Bangia' sp*. OUCPT-01 in the Bangiales

Min Cao[1,3], Guiqi Bi[1,3], Yunxiang Mao[1,2,3*], Guiyang Li[4], Fanna Kong[1,3].
1Key Laboratory of Marine Genetics and Breeding (OUC), Ministry of Education, Qingdao, P.R. China; 2Laboratory for Marine Biology and Biotechnology, Qingdao National Laboratory for Marine Science and Technology, Qingdao, China; 3College of Marine Life Sciences, Ocean University of China, Qingdao, and 4Key Laboratory for Sustainable Utilization of Marine Fisheries Resources, Ministry of Agriculture, Yellow Sea Fisheries Research Institute, Chinese Academy of Fishery Sciences, Qingdao, China. 266003.
*Author for correspondence: Yunxiang Mao
E-mail address: yxmao@ouc.edu.cn. Tel: +86 532 82032017 (Office).
ORCID of correspondence anthor: 0000-0003-2432-4643

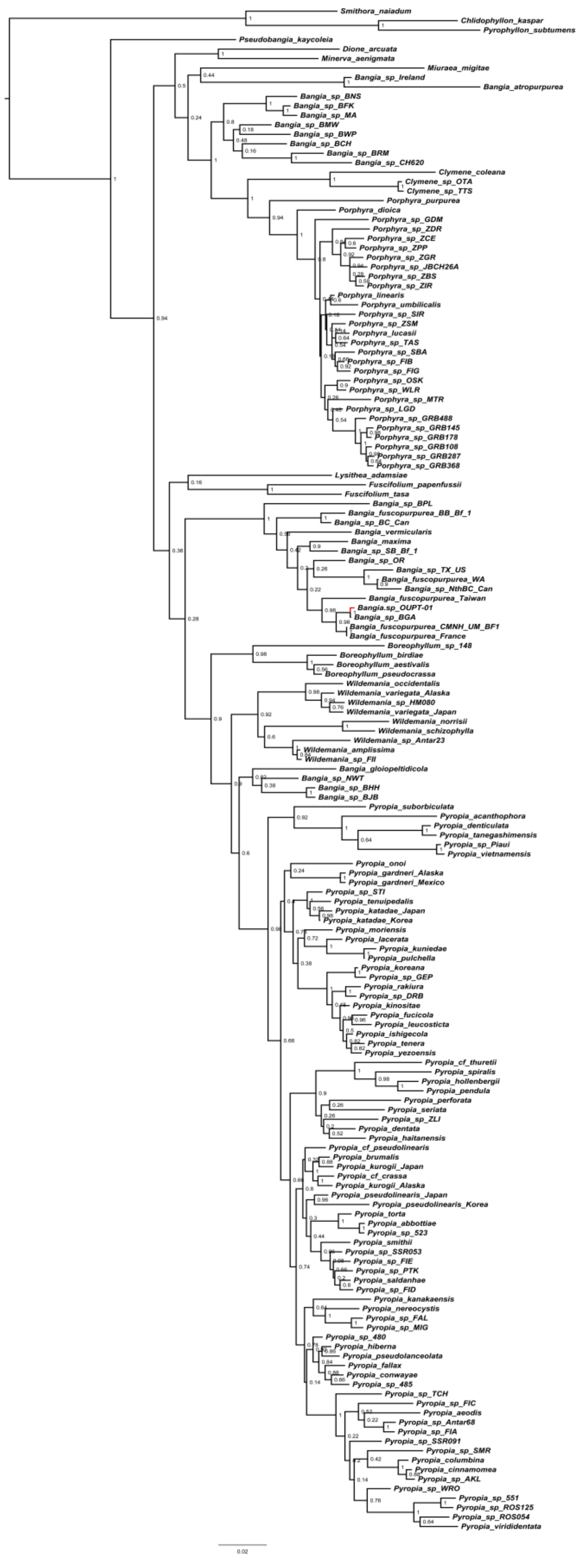

Supporting Information Figure S1 Maximum likelihood phylogenetic tree based on concatenated rbcL and nrSSU genes

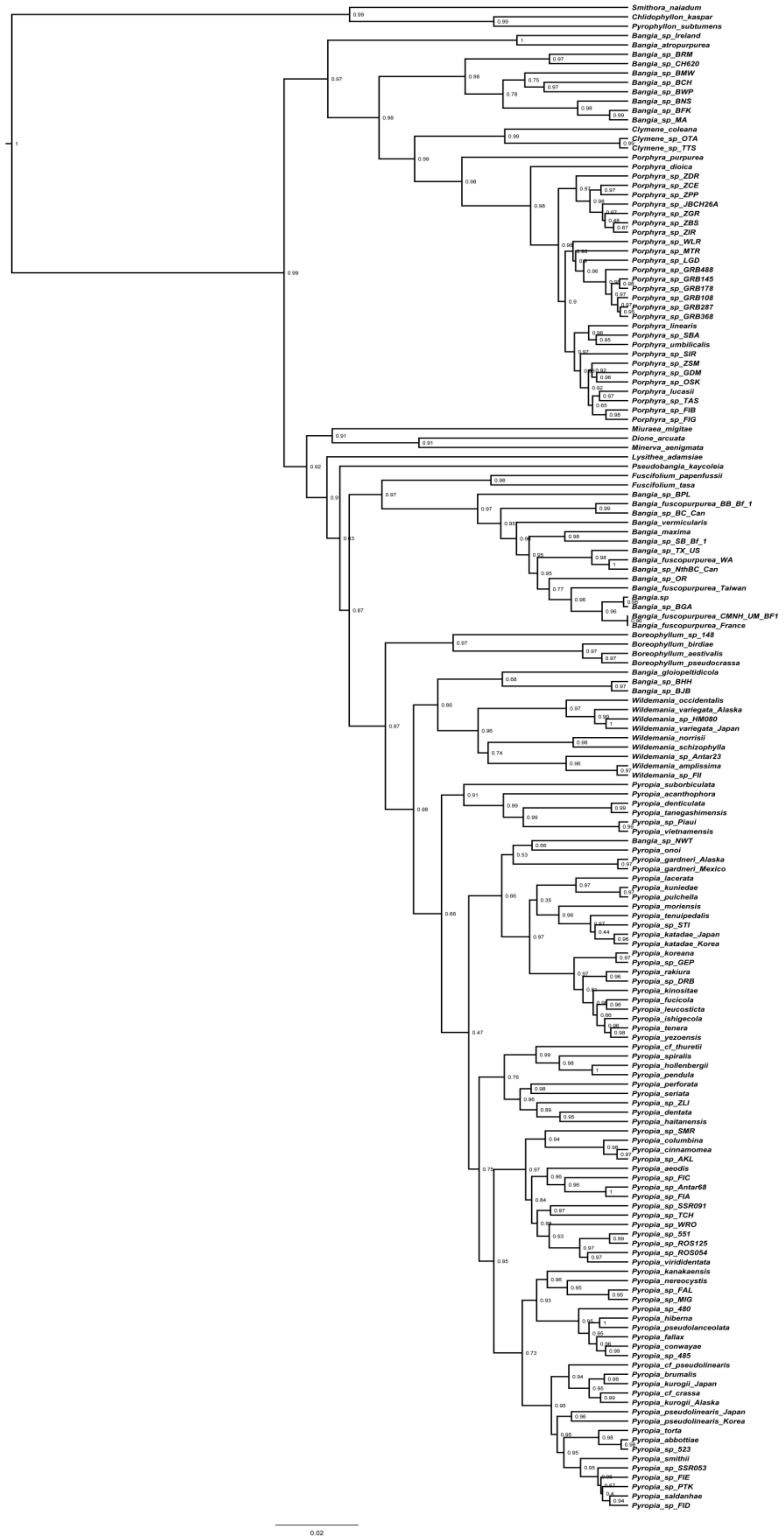

Supporting Information Figure S2 Bayesian phylogenetic tree based on concatenated r*bcL* and nrSSU genes

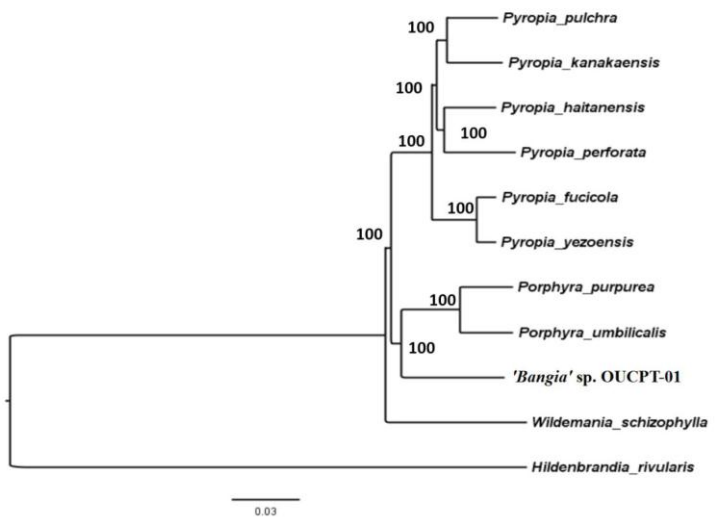

Supporting Information Figure S3 Maximum likelihood phylogenetic tree based on 153 common genes in the Bangiales(for amino acid dataset)

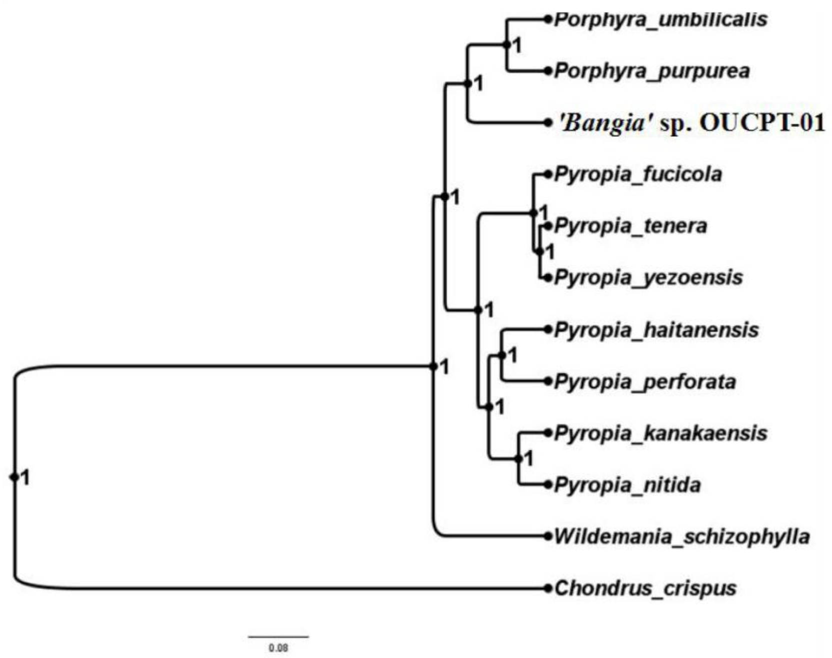

Supporting Information Figure S4 Phylogenetic tree based on 18 concatenated mitochondrial gene

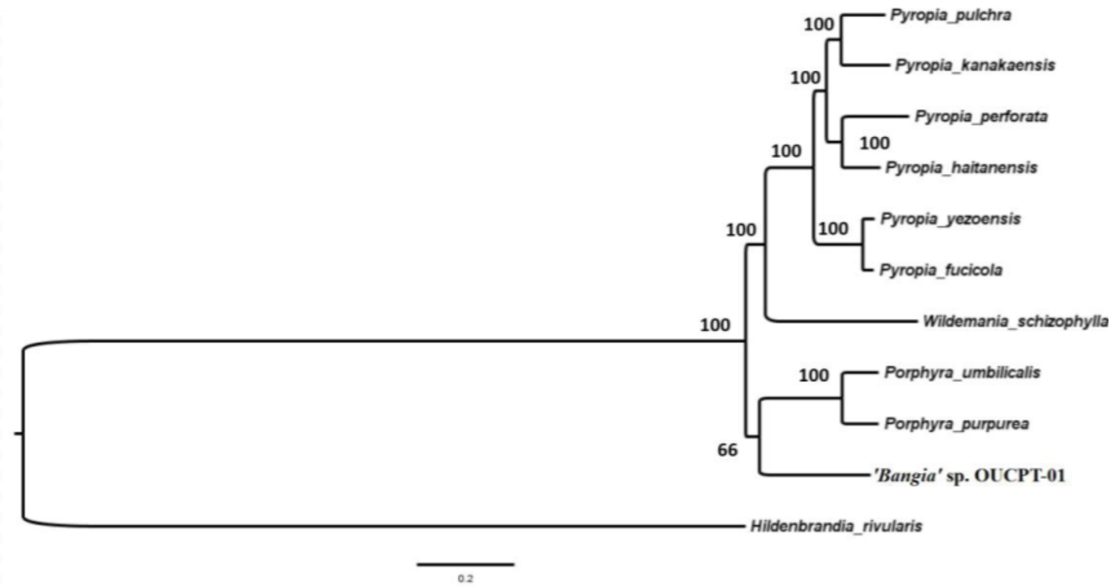

Supporting Information Figure S5 Maximum likelihood phylogenetic tree based on 153 common genes in the Bangiales(for nuclei acid dataset)

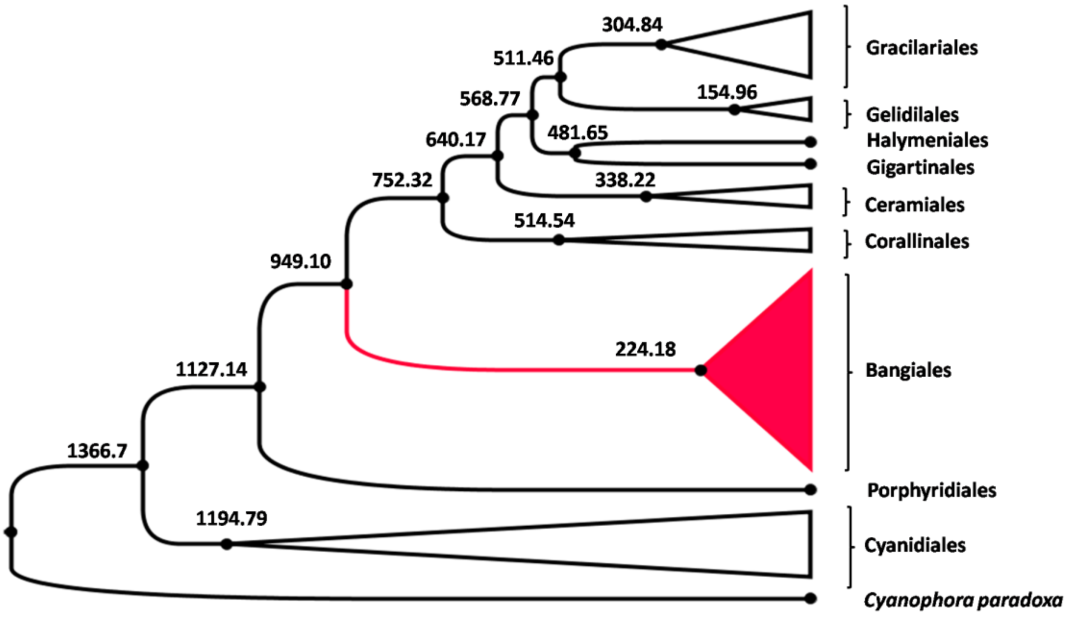

Supporting Information Figure S6 Chronogram showing estimated divergence times among Rhodophyta by Bayesian

Supporting Information Table S1 List of genes found in the *'Bangia' sp.* OUCPT-01 plastid genome

RNA genes								
	tRNAs	trnH-GTG	trnI-GAT	trnW-CCA	trnI-CAT	trnS-GCT	trnY-GTA	trnA-GGC
		trnS-TGA	trnP-TGG	trnG-TCC	trnR-TCT	trnN-GTT	trnT-GGT	trnD-GTC
		trnT-TGT	trnV-GAC	trnI-GAT	trnE-TTC	trnL-TAA	trnR-CCT	trnL-GAG
		trnM-CAT	trnA-TGC	trnL-TAG	trnL-CAA	trnF-GAA	trnR-CCG	trnQ-TTG
		trnV-TAC	trnS-CGA	trnG-GCC	trnS-GGA	trnfM-CAT	trnR-ACG	trnC-GCA
		trnK-TTT	trnA-TGC					
	rRNAs	*rrfA*	*rrfB*	*rrlA*	*rrlB*	*rrsA*	*rrsB*	
Photosynthesis genes								
Acetyl-coa carboxylase			*accA*	*accB*	*accD*	*fabH*	*acpP*	
ATP-dependent protease			*clpC*					
ATP synthase		*atpA*	*atpB*	*atpD*	*atpE*	*atpF*	*atpH*	*atpI*
		atpG						
Cytochrome b/f		*petA*	*petB*	*petD*	*petF*	*petG*	*petN*	*petJ*
Cytochrome c biogenesis			*ccsA*	*ccs1*				
Photosystem I		*psaA*	*psaB*	*psaC*	*psaD*	*psaE*	*psaF*	*psaI*
		psaL	*psaM*	*psaJ*	*psaK*			
Photosystem II		*psbA*	*psbC*	*psbD*	*psbE*	*psbF*	*psbJ*	*psbH*
		psbI	*psbK*	*psbL*	*psbN*	*psbT*	*psbV*	*psbW*
		psbX	*psbY*	*psbZ*	*psbB*			
Rubisco		*rbcL*	*rbcR*	*rbcS*	*odpB*	*odpA*		
Translation.initiation factor			*infB*	*infC*	*tufA*	*tsf*		
Membrane protein		*cemA*	*secY*	*secA*				
Amino acids		*trpA*	*trpG*	*argB*	*gltB*	*ilvH*	*ilvB*	
RNA polymerase		*rpoA*	*rpoB*	*rpoC1*	*rpoC2*			
DNA replication		*dnaK*	*dnaB*					
Organelle division		*ftsH*	*groEL*					
Components of phycobilisomes			*cpeA*	*apcA*	*apcB*	*apcD*	*apcE*	*apcF*
			cpeB	*cpcA*	*cpcB*	*cpcG*		
Nucleotides		*carA*						
Redox system		*trxA*	*pbsA*	*bas1*				
Cofactors		*preA*	*chlL*	*chlN*	*chlB*	*chlI*		
Ribosomal proteins								

		rpl1	*rpl2*	*rpl3*	*rpl4*	*rpl5*	*rpl9*	*rpl14*
		rpl11	*rpl2*	*rpl13*	*rpl16*	*rpl18*	*rpl9*	*rpl28*
		rpl20	*rpl21*	*rpl22*	*rpl23*	*rpl24*	*rpl27*	
		rpl29	*rpl31*	*rpl32*	*rpl33*	*rpl34*	*rpl35*	
Large subunit		*rpl36*	*rpl6*					
		rps1	*rps2*	*rps3*	*rps4*	*rps5*	*rps6*	*rps20*
		rps7	*rps8*	*rps9*	*rps10*	*rps11*	*rps12*	
Small subunit		*rps13*	*rps14*	*rps16*	*rps17*	*rps18*	*rps19*	
Other genes		*cbbx*	*sufC*	*glnK*	*tatC*			
ORFs		*orf17*	*orf27*	*orf287*	*orf293*	*orf621*	*orf68*	*orf108*
		orf71	*orf75*	*orf107*	*orf111*	*orf114*	*orf121*	*orf240*
		orf148	*orf149*	*orf174*	*orf198*	*orf203*	*orf238*	*orf263*
		orf320	*orf327*	*orf382*	*orf38*	*orf450*	*orf565*	*ycf12*
		ycf3	*ycf17*	*ycf19*	*ycf20*	*ycf21*	*ycf22*	*ycf23*
		ycf24	*ycf26*	*ycf28*	*ycf29*	*ycf33*	*ycf34*	*ycf35*
		ycf36	*ycf37*	*ycf38*	*ycf39*	*ycf4*	*ycf46*	*ycf61*
		ycf18	*ycf59*	*syh*	*thiG*	*ftrC*	*pgmA*	*rne*
		syfB						

Supporting Information Table S2 The gene contents in Venn diagram

Names	Total	Elements							
B.txt<br>PH.txt<br>PU.txt<br>WS.txt	187	*rpl5*	*orf108*	*psbK*	*rpl20*	*orf198*	*petF*	*clpC*	*psaF*
		atpF	*psbE*	*psbW*	*atpH*	*fabH*	*ilvH*	*trpG*	*ilvB*
		rpl4	*rps7*	*groEL*	*ycf33*	*rps14*	*petD*	*orf107*	*psbV*
		psaK	*atpA*	*ycf59*	*dnaB*	*psbI*	*chlN*	*psbD*	*rps16*
		rpl3	*rpl27*	*cemA*	*csA*	*apcE*	*rpl19*	*cpcG*	*orf121*
		orf238	*thiG*	*gltB*	*orf148*	*rbcL*	*infB*	*ycf39*	*ftsH*
		ycf3	*dnaK*	*orf203*	*rpl32*	*secA*	*carA*	*cpcA*	*rpl14*
		psaC	*rpl22*	*rbcR*	*orf75*	*psaD*	*psaI*	*rps18*	*rpl1*
		orf240	*rpl33*	*psbL*	*ccs1*	*cpcB*	*rpl23*	*ycf61*	*rpoB*
		rpl36	*psbC*	*psaM*	*ycf35*	*ycf28*	*rpl18*	*rps11*	*rps4*
		petA	*rps6*	*ycf22*	*accB*	*rps9*	*rps2*	*cpeA*	*ycf46*
		accD	*chlI*	*psbF*	*petN*	*rpl16*	*rpl28*	*argB*	*syh*
		rpl12	*psbT*	*orf71*	*apcD*	*orf565*	*petB*	*pgmA*	*rpl6*
		petG	*pbsA*	*tufA*	*ycf21*	*chlL*	*rps12*	*rpl2*	*rps3*
		psaE	*chlB*	*atpB*	*apcF*	*rps10*	*rps1*	*psaL*	*psaA*
		orf450	*apcA*	*glnB*	*rps5*	*atpG*	*psbX*	*cbbX*	*orf263*
		rpoC1	*rpl11*	*accA*	*atpE*	*psbN*	*trpA*	*atpD*	*ycf37*
		rpl135	*rpl21*	*orf174*	*ycf34*	*preA*	*psaJ*	*cpeB*	*rpl13*
		rps19	*trxA*	*ycf29*	*rpl31*	*syfB*	*ycf23*	*psbJ*	*odpA*
		ycf20	*ycf26*	*rpl34*	*rps8*	*ycf38*	*apcB*	*rsp20*	*tatC*
		ycf17	*rpl9*	*psbH*	*orf320*	*orf327*	*ycf36*	*rpoC2*	*ycf19*
		ycf12	*atpI*	*rpoA*	*rps17*	*petJ*	*odpB*	*psbZ*	*rbcS*
		secY	*rps13*	*infC*	*rpl24*	*tsf*	*psaB*	*rne*	*ycf46*
		psbA	*acpP*	*orf`149*					
B.txt<br>PH.txt<br>PU.txt	3	*ycf24*	*ycf18*	*psbB*					
B.txt<br>PH.txt<br>WS.txt	7	*orf621*	*orf68*	*orf382*	*rpl29*	*orf287*	*orf111*	*orf114*	
PH.txt<br>PU.txt<br>WS.txt	5	*orf62*	*ycf65*	*ycf31*	*orf58*	*ftrB*			
B.txt<br>PH.txt	1	*orf27*							
B.txt<br>WS.txt	3	*psbY*	*sufC*	*bas1*					

PH.txt PU.txt	4	*orf199*	*ycf16*	*ycf7*	*ycf32*				
PH.txt WS.txt	1	*orf36*							
B.txt	2	*ftrC*	*orf38*						
PH.txt	1	*orf32*							
PU.txt	8	*orf263a*	*orf288*	*orf110*	*orf243*	*orf71a*	*orf65*	*orf110a*	*orf383*
WS.txt	7	*ycf63*	*sufB*	*moeB*	*psi*	*ompR*	*petL*	*nblA*	

Supporting Information Table S3 The nrSSU and *rbc*L sequences for phylogenetic analysis

	GenBank Accession No.	
Samples	nrSSU	*rbcL*
'Bangia' sp. OUCPT-01	KP747608	KP747609
Bangia atropurpurea	AF169341	AF169330
Bangia sp. Ireland	AF043365	AF043371
'Bangia fuscopurpurea' BB Bf 1	EU289023	EU289018
'Bangia fuscopurpurea' CMNH UM BF1	HQ687561	HQ687502
'Bangia fuscopurpurea' France	AF175535	AF168659
'Bangia fuscopurpurea' Taiwan	AF175529	AF168654
'Bangia fuscopurpurea' WA	AF169336	AF169329
'Bangia' gloiopeltidicola	HQ687563	HQ687503
'Bangia' maxima	EU289025	EU289020
'Bangia' sp. BC Can	AF043359	AF043376
'Bangia' sp. BCH	AY184335	HQ687504
'Bangia' sp. BFK	AY184338	HQ687505
'Bangia' sp. BGA	AY184341	HQ687506
'Bangia' sp. BHH	AY184339, AY184340	GU046404
'Bangia' sp. BJB	AY184337	HQ687507
'Bangia' sp. BMW	AY184344	HQ687508
'Bangia' sp. BNS	AY184345	HQ687509
'Bangia' sp. BPL	DQ084436 & DQ084437	HQ687510
'Bangia' sp. BRM	HQ687562	HQ687511
'Bangia' sp. BWP	AY184348	EU570051

'Bangia' sp. CH620	HQ728195	HQ728203
'Bangia' sp. MA	AF043362	AF043369
'Bangia' sp. NthBC Can	AF043360	AF043372
'Bangia' sp. NWT	AF043355	AF043366
'Bangia' sp. OR	AF043358	AF043367
'Bangia' sp. SB Bf 1	EU289024	EU289019
'Bangia' sp. TX	AF043361	AF043377
'Bangia' vermicularis	EU289027	EU289022
Boreophyllum aestivale	GU319836	EU223033
Boreophyllum birdiae	HQ709388	AY180909
Boreophyllum pseudocrassum	HQ687564	HQ687512
Boreophyllum sp. 148	GU319852	EU223240
Chlidophyllon kaspar	AY126431	HQ687513
Clymene coleana	AF136423	FJ263672
Clymene sp. OTA	GU214024	GU214023
Clymene sp. TTS	HQ687565	HQ687514
Dione arcuata	AY465354	EU570052
Fuscifolium papenfussii	GU319855	EU223120
Fuscifolium tasa	GU319862	EU223226
Lysithea adamsiae	HQ687566	HQ687515
Minerva aenigmata	AY465355	EU570053
Miuraea migitae	EU521642	EU521643
Porphyra dioica	HQ687579	HQ687546
Porphyra linearis	HQ687580	HQ687547
Porphyra lucasii	AY139685	AY139687
Porphyra purpurea	HQ687567	HQ687516
Porphyra sp. FIB	AY909598	GU165840
Porphyra sp. FIG	GU165881	GU165885
Porphyra sp. GDM	AY909597	GU046415
Porphyra sp. GRB108	AF136420	GU214021
Porphyra sp. GRB145	AY184349	HQ687548
Porphyra sp. GRB178	AY909603	HQ687549
Porphyra sp. GRB287	AY909595	HQ687550
Porphyra sp. GRB368	AY292639	HQ687551
Porphyra sp. GRB488	AY184350	GU046405
Porphyra sp. JBCH26A	HQ687581	HQ687552
Porphyra sp. LGD	AF136422	GU046409
Porphyra sp. MTR	HQ687582	HQ687553
Porphyra sp. OSK	AY909593	HQ687554
Porphyra sp. SBA	AY909589	GU046414

Porphyra sp. SIR	AY909588	GU046417
Porphyra sp. TAS	AY909585	GU046427
Porphyra sp. WLR	AY292644 AY292645	GU165837
Porphyra sp. ZBS	AY292626	HQ687555
Porphyra sp. ZCE	AY292627	GU046424
Porphyra sp. ZDR	AY292628	GU046425
Porphyra sp. ZGR	AY292631	HQ687556
Porphyra sp. ZIR	AY292632	GU214022
Porphyra sp. ZPP	AY292636	HQ687557
Porphyra sp. ZSM	HQ687583	HQ687558
Porphyra umbilicalis	HQ687584	HQ687559
Pseudobangia kaycoleia	AF043364	-
Pyrophyllon subtumens	HQ687568	HQ687517
Pyropia abbottiae	GU319835	EU223024
Pyropia acanthophora	L26197	HQ605695
Pyropia aeodis	AY292624, AY292625	GU165843
Pyropia brumalis	GU319837	EU223038
Pyropia cf *crassa*	HQ687569	HQ687518
Pyropia cf *pseudolinearis*	GU319858	EU223172
Pyropia cf *thuretii*	HQ687587	HQ687519
Pyropia cinnamomea	AH008010	EU521637
Pyropia columbina	GU046398	GU046423
Pyropia conwayae	GU319838	EU223045
Pyropia dentata	HQ687588	HQ687520
Pyropia denticulata	HQ687570	HQ687521
Pyropia fallax	GU319840	GU319865
Pyropia fucicola	GU319841	EU223088
Pyropia gardneri AK	GU319842	EU223096
Pyropia gardneri Mexico	DQ084423	HQ687522
Pyropia haitanensis	AB013181	AB118585
Pyropia hiberna	GU319843	GU319866
Pyropia hollenbergii	HQ687589	HQ687523
Pyropia ishigecola	HQ687571	HQ687524
Pyropia kanakaensis	GU319844	EU223099
Pyropia katadae Japan	HQ687572	HQ687525
Pyropia katadae Korea	HQ728191	HQ728199
Pyropia kinositae	EU521640	EU521641
Pyropia koreana	HQ728190	HQ728198
Pyropia kuniedae	HQ728192	HQ728200
Pyropia kurogii AK	GU319845	EU223105

Pyropia kurogii Japan	HQ687573	HQ687526
Pyropia lacerata	HQ687574	HQ687527
Pyropia leucosticta	HQ687593	HQ687528
Pyropia moriensis	EU521644	EU521645
Pyropia nereocystis	GU319849	EU223117
Pyropia onoi	HQ687575	HQ687529
Pyropia pendula	DQ084430	HQ687530
Pyropia perforata	AY909592	EU223127
Pyropia pseudolanceolata	GU319857	EU223145
Pyropia pseudolinearis Japan	HQ687590	HQ687531
Pyropia pseudolinearis Korea	HQ728188	HQ728196
Pyropia pulchella	HQ687591	HQ687532
Pyropia rakiura	AF136425	EU521646
Pyropia saldanhae	AY292630	GU165838
Pyropia seriata	HQ687576	HQ687533
Pyropia smithii	GU319861	EU223224
Pyropia sp. 480	GU319846	GU319867
Pyropia sp. 485	GU319847	GU319868
Pyropia sp. 523	GU319853	GU319869
Pyropia sp. 551	GU319854	GU319870
Pyropia sp. AKL	GU046402	GU046403
Pyropia sp. Antar68	HQ605699	HQ605698
Pyropia sp. DRB	AY909599	HQ687534
Pyropia sp. FAL	DQ084424, DQ084425	HQ687535
Pyropia sp. FIA	AY292637	GU165842
Pyropia sp. FIC	AY292638	GU046422
Pyropia sp. FID	GU046396	GU046406
Pyropia sp. FIE	AH015106	GU046408
Pyropia sp. GEP	AY909596	GU165841
Pyropia sp. MIG	DQ084426, DQ084427	HQ687536
Pyropia sp. Piaui	AY766357	HQ605697
Pyropia sp. PTK	HQ687592	HQ687537
Pyropia sp. ROS054	AF136426	GU046410
Pyropia sp. ROS125	AY184352 & AY184353	HQ687538
Pyropia sp. SMR	AY909587	HQ687539
Pyropia sp. SSR053	AF136427	GU046411
Pyropia sp. SSR091	AF136428	GU046421
Pyropia sp. STI	AY909584	HQ687540
Pyropia sp. TCH	AY909583	GU046418

Pyropia sp. WRO	AY909586	HQ687541
Pyropia sp. ZLI	AY292634, AY292635	GU165839
Pyropia spiralis	AY766360	HQ605696
Pyropia suborbiculata	HQ728193	HQ728201
Pyropia tanegashimensis	HQ727887	HQ687542
Pyropia tenera	HQ687577	HQ687543
Pyropia tenuipedalis	EU521648	EU521649
Pyropia torta	GU319863	EU223236
Pyropia vietnamensis	HQ687578	HQ687544
Pyropia virididentata	AF136421	EU521650
Pyropia yezoensis	HQ728189	HQ728197
Smithora naiadum	AF087129	HQ687545
Wildemania amplissima	HQ687585	HQ687560
Wildemania norrisii	GU319850	EU223212
Wildemania occidentalis	GU319851	EU223118
Wildemania schizophylla	GU319860	GU319871
Wildemania sp. Antar23	HQ605701	HQ605700
Wildemania sp. FII	GU165844	GU165883
Wildemania sp. HM080	HQ728194	HQ728202
Wildemania variegata AK	GU319864	EU223237
Wildemania variegata Japan	GU046401	GU046430

Supporting Information Table S4 PCR primers used to vertify the accuracy of the plastid genome

Number	Primer sequence	PCR product size (bp)
1	5' AATGATAGGATACCGAGAC3'<br>5' ACAGGTGCTGTTGATAAG 3'	1123
2	5'AGAGCAATAAACCGAAGT3'<br>5' ATAGGTCCCTGAGCGATA3'	1808
3	5'GCAGTCAGAGCAAATGGGTA3'<br>5' GCGTGGTGGAATTGGTAGA3'	1320
4	5'TCAGAGCGGCGTTATACAC3'<br>5'CACAAGTTTGGCTGGCATT3'	1450
5	5' TCCAGGTAGATCACCCA3'<br>5'ATTAGCAGGAGAAGCACC3'	2012
6	5'GAAGGCGAACAGTTAGGT3'<br>5'TCGAAAGCATGGGAATAG3'	2089

7	5' CGCTGGAACTGCTGAACT3'<br>5' ATCAATGTCCTGTGCCTC3'	2760
8	5'TAACCCAGATTTGACCAT3'<br>5'TATACCGCCAGTACCCTA3'	1671
9	5'ACGAGCTGTCATTAGACC3'<br>5'CAAAGTAACGGACGCATA3'	2581
10	5'GAGAAGACCTGCGACATT3'<br>5' ATTTGGTCCTGCCTCTAA3'	2690
11	5'TTCCCAGTCATCTTAGTCG3'<br>5'ATTACCCTCAAATCCATCC3'	2583
12	5'TTTTAGGTAGAGGAATGGGAGG3'<br>5' ATCAGCGGCATGAGAAGC 3'	2352
13	5'AACGCTTGCGATTACAGG3'<br>5'AAGGGATTGGTGTTGAGC3'	2492
14	5'TTACGAAAGCACTTGCCTCT3'<br>5' CAACTATTTACCTCCTCAC3'	2632
15	5'ACTAATGATGTCGCTGGTG3'<br>5'TGATGCTTGGGAAGAACTA3'	962
16	5'AAGCCAGATGATTGTAACCG3'<br>5'GTAGTTTGGACGCAAGAGCA3'	1999
17	5'AAGTGCCTTCGGAATGTG3'<br>5' GTTGGCTTAATGGTTTGC3'	1155
18	5'TACACTAATCGTCGTATGG3'<br>5'AGAGGTAGAGGCACAAAA3'	2947
19	5'CTTTCACGGAGGTAACGG3'<br>5'TGGAATTGTAGGCCAGCT3'	2265
20	5'TAGATTCTCCTGTCACCCAT3'<br>5'CTGAACATAGTCCACGCTTA3'	2540
21	5'TAAACTAGAGCCAAGGAA3'<br>5'AGACTGGAGCAGGTATGA3'	2644
22	5'TGCCTTCTGCTGTTGGTTAT3'<br>5'TCTTTGTATTGGGAGCCTTT3'	2066
23	5' ATAGTTGTGGTACGGGTTTCT 3'<br>5' TATTTGGTCAGGCAATCATCC 3'	1867
24	5'CATTTCCTGCGACCTGTTCC3'<br>5'CAGTAGCGTTGATTCCGTTT3'	1543
25	5'TTCTATACCTTCGCCCATTT3'<br>5'TCGTCAAGCCCATACTACAT3'	2419
26	5' AACCGTAACAAATGCTCCT 3'<br>5' GCCGTAGTTCTAAATCCCT 3'	2449
27	5' TTCAGTTGGTAGAACGCAGGTC 3'<br>5' GAGGCGGTGTTAGCGTAGGT 3'	2585

28	5'CTTGAGCACCTTGTTGTA3'<br>5' GAGTCATTCCCTTTCTTT3'	2413
29	5' TCGGTTGTGCTACAGTTA 3'<br>5' TTGGAGGAGTTACGAATA 3'	2242
30	5' TCCACCGAAAGGAAGACTGA3'<br>5'TTGACTTGATGAGCGACGAA 3'	2634
31	5'CACCTGGACAGAAAGACCCT 3'<br>5'AACCACCACAAACAACATCACC 3'	1507

Supporting Information Table S5 Gene components of 153 common genes and 67 common genes for Bangiales and Rhodophyta

Gene components of 153 common genes for Bangiales									
accA	*accB*	*accD*	*acpP*	*acsF*	*apcA*	*apcB*	*apcD*	*apcE*	*apcF*
atpA	*atpB*	*atpD*	*atpE*	*atpF*	*atpG*	*atpH*	*atpI*	*bas1*	*carA*
cbbX	*ccs1*	*cemA*	*chIL*	*cpcA*	*cpcB*	*cpcG*	*cpeA*	*cpeB*	*csA*
dnaK	*fabH*	*ftrB*	*gltB*	*groEL*	*ilvH*	*infB*	*infC*	*odpA*	*odpB*
petA	*petB*	*petD*	*petE*	*petF*	*petG*	*petJ*	*petN*	*pgmA*	*preA*
psaB	*psaC*	*psaD*	*psaF*	*psaI*	*psaI*	*psaJ*	*psaK*	*psaM*	*psb28*
psbA	*psbA*	*psbB*	*psbC*	*psbD*	*psbE*	*psbF*	*psbH*	*psbI*	*psbK*
psbL	*psbN*	*psbT*	*psbV*	*psbZ*	*rbcL*	*rbcR*	*rbcS*	*rne*	*rp13*
rpl1	*rpl11*	*rpl12*	*rpl14*	*rpl16*	*rpl18*	*rpl19*	*rpl2*	*rpl20*	*rpl21*
rpl22	*rpl23*	*rpl27*	*rpl28*	*rpl3*	*rpl31*	*rpl33*	*rpl36*	*rpl4*	*rpl5*
rpl6	*rpoA*	*rpoB*	*rpoC1*	*rpoC2*	*rpoZ*	*rps1*	*rps10*	*rps11*	*rps12*
rps13	*rps14*	*rps16*	*rps17*	*rps19*	*rps2*	*rps20*	*rps3*	*rps4*	*rps5*
rps6	*rps7*	*rps8*	*rps9*	*secA*	*secY*	*sufB*	*sufC*	*syh*	*tatC*
thiG	*tilS*	*trpA*	*trpG*	*trxA*	*tsf*	*tufA*	*ycf19*	*ycf29*	*ycf3*
ycf36	*ycf38*	*ycf39*	*ycf4*	*ycf45*	*ycf52*	*ycf53*	*ycf54*	*ycf55*	*ycf61*
ycf63	*ycf65*	*ycf80*							
Gene components of 67 common genes for Rhodophyta									
apcA	*apcB*	*apcD*	*apcE*	*apcF*	*atpA*	*atpB*	*atpE*	*atpG*	*chIL*
petA	*petB*	*petD*	*petF*	*preA*	*psaC*	*psaF*	*psbA*	*psbB*	*psbC*
psbD	*psbE*	*psbF*	*psbH*	*psbK*	*psbL*	*psbN*	*psbT*	*psbV*	*psbW*
rbcL	*rbcR*	*rpl1*	*rpl2*	*rpl3*	*rpl5*	*rpl6*	*rpl11*	*rpl12*	*rpl14*
rpl16	*rpl18*	*rpl20*	*rpl21*	*rpl22*	*rpl33*	*rpoB*	*rps2*	*rps3*	*rps4*
rps5	*rps7*	*rps8*	*rps9*	*rps10*	*rps11*	*rps12*	*rps14*	*rps16*	*rps17*
rps19	*secY*	*tufA*	*ycf3*	*ycf4*	*ycf24*	*ycf39*			

Supporting Information Table S6 Plastid genomes used for gene content comparisons and phylogenetic studies

Species	Accession Number	Date
'Bangia' sp. OUCPT-01	KP 714733	this study
Cyanophora paradoxa	NC_001675.1	04/15/2009
Cyanidioschyzon merolae	NC_004799	04/15/2009
Calliarthron tuberculosum	NC_021075	05/17/2013
Chondrus crispus	NC_020795	04/05/2013
Cyanidium caldarium	NC_001840	05/06/2009
Cyanidiaceae sp.	KJ569775	03/12/2014
Gracilaria tenuistipitata	NC_006137	08/13/2004
Gracilaria salicornia	NC_023785	03/20/2014
Grateloupia taiwanensis	NC_021618	08/06/2013
Galdieria sulphuraria	NC_024665	08/06/2014
Gelidium elegans	NC_029858.1	04/12/2016
Gelidium vagum	NC_029859.1	04/12/2016
Gracilaria chilensis	NC_029860.1	04/12/2016
Gracilariopsis lemaneiformis	NC_029644.1	03/11/2016
Laurencia sp.	LN833431.1	06/01/2015
Porphyra purpurea	NC_000925	03/26/2010
Porphyra umbilicalis	JQ408795.	19/09/2012
Porphyridium purpureum	NC_023133	12/20/2013
Pyropia haitanensis	NC_021189	05/16/2013
Pyropia fucicola	KJ776837	04/06/2014
Pyropia kanakaensis	KJ776836	04/06/2014
Pyropia perforate	NC_024050	04/06/2014
Pyropia yezoensis	KC517072	05/16/2013
Sporolithon durum	NC_029857.1	04/12/2016
Vertebrata_lanosa	KP308097.1	07/26/2016
Wildemania schizophylla	NC_029576	03/03/2016
